# A Rare Case of Chondrosarcoma With Metastasis to the Oral Cavity

**DOI:** 10.7759/cureus.16283

**Published:** 2021-07-09

**Authors:** Amitabh Kumar Upadhyay, Aaditya Prakash, Farah Rana, Neeraj Jain

**Affiliations:** 1 Medical Oncology, Tata Main Hospital, Jamshedpur, IND; 2 Radiation Oncology, Tata Main Hospital, Jamshedpur, IND; 3 Pathology, Tata Main Hospital, Jamshedpur, IND

**Keywords:** chondrosarcoma, rare, metastasis, oral, presentation

## Abstract

Chondrosarcomas with metastases to oral cavities are extremely rare. To the best of our knowledge, only six cases of chondrosarcoma with metastases to the oral cavity, maxilla, and/or mandible have been reported in the English-language literature. The last such case was reported three decades earlier; none of the reported cases were from India. We present the case of an Indian patient with chondrosarcoma in the knee region, who was treated with surgical excision in 2013. However, he experienced a recurrence in 2019, developed upper gingival metastasis in 2020, and succumbed within two months of this unique presentation. Oral metastatic lesions have a wide differential diagnosis, and awareness of this rare presentation can help clinicians maintain an index of suspicion for an underlying metastatic malignancy. Our experience emphasizes the importance of detailed history-taking, clinical examination, and consideration of metastases as a differential diagnosis, even if there is no reported history of malignancy. Such lesions may also be the first sign of an occult primary tumor, which will require prompt investigation for early diagnosis and treatment.

## Introduction

Metastases to the oral cavity are rare and usually occur from melanomas and cancers of the breast, lung, kidney, and liver [1-4]. To the best of our knowledge, only six cases of chondrosarcoma with metastases to the oral cavity, maxilla, and/or mandible have been reported in the literature, with the last case having been reported three decades earlier in 1991 [5-10]. Metastasis to the gingiva or to soft tissue only has been reported in three patients [8-10], although no such cases have been reported in India. Herein, we present the case of an Indian patient with a history of conventional chondrosarcoma, who developed recurrent chondrosarcoma with metastasis to the upper gingivobuccal sulcus, four years after surgical resection of the primary tumor from the lower right femur, and we review the relevant literature on this rare clinical presentation.

## Case presentation

**Figure 1:**
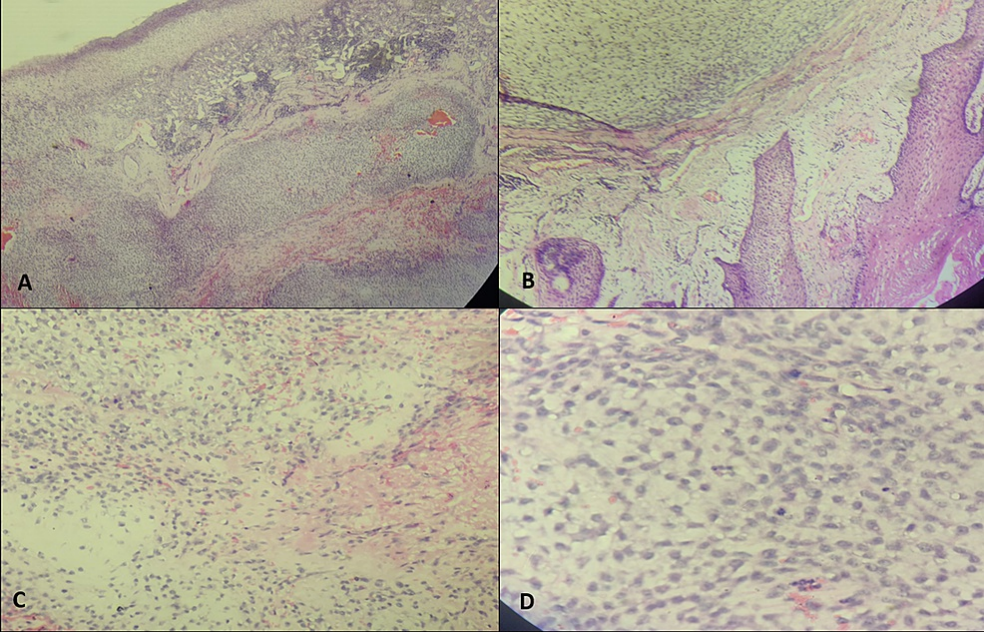
Haematoxylin and Eosin stained histomorphology images of the excised alveolar lesion. A (40x) & B (100x) - Ulcerated surface epithelium with underlying proliferated capillaries and lobules of tumor cells in a chondromyxoid background. C (200x) & D (400x) - Uniform tumor cells with pale eosinophilic to vacuolated cytoplasm and few atypical mitotic figures.

A 52-year-old man with complaints of swelling and pain in the right lower thigh was evaluated and diagnosed with a locally advanced chondrosarcoma, for which he underwent right distal femoral excision and replacement with a mega prosthesis in February 2013. Tumor histopathology was suggestive of conventional chondrosarcoma, Grade 1. The patient was followed up regularly. In April 2019, he presented with a huge, circumferential, bony swelling at the distal end of the femur on the right side. The upper limit of the swelling was ~10 cm below the inguinal crease and was associated with a flexion deformity of the leg. Thoracoabdominal and right thigh contrast-enhanced computed tomography (CT) showed enhanced soft tissue lesions in bilateral basal lung fields with the largest in the left lower lobe, measuring 30 × 34 × 40 mm, suggestive of metastasis, along with a large 13 × 12 × 6 cm soft tissue mass at the anterior aspect of the distal part of the right thigh, involving the quadriceps. The case was discussed by the institutional Tumor Board, and amputation was offered as a treatment alternative to the patient. The patient refused amputation and opted for palliative radiation, which was completed in May 2019. The patient was temporarily lost to follow-up; he presented again in October 2020 with increased swelling and pain in the right thigh. He also complained of proliferative soft tissue growth in the upper gingivobuccal sulcus, though he did not reveal his history of chondrosarcoma. An excision biopsy was performed considering a benign differential diagnosis; pathological examination showed neoplastic cells against a loose myxoid background with overall features suggestive of a bone-forming neoplasm, possibly chondrosarcoma (Figure 1).

**Figure 2:**
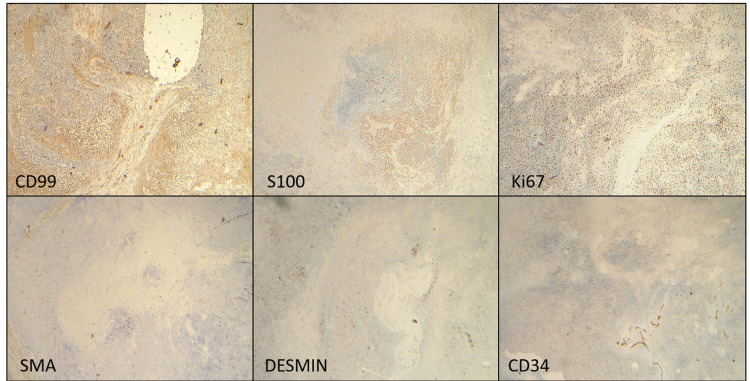
Immunohistochemistry on the tumor sections. Neoplastic cells are positive for CD99 (upper left), S100 ( upper central) and show 60% Ki67 index (upper right). The tumor cells are negative for SMA, Desmin, CD34 (shown in lower panels), and CK (not shown here)– favoring a diagnosis of myxoid chondrosarcoma

Immunohistochemically, the tumor cells were positive for the expression of S100 and CD99 and negative for the expression of SMA, CD34, CK, and Desmin, with 60% of cells positive for the expression of Ki67 (Figure 2).

**Figure 3:**
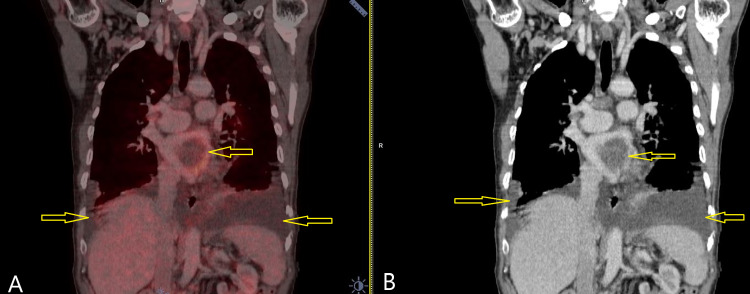
Nodules in bilateral lung fields with bilateral moderate pleural effusion and thrombus in left atrium extending into pulmonary veins. Seen with Fluorodeoxyglucose (FDG) avidity in fused PET-CT (A) as well as in contrast CT (B).

A whole-body positron emission tomography-computer tomography (PET-CT) was performed after the excision biopsy of the gingivobuccal lesion; it showed mild fluorodeoxyglucose (FDG) uptake along right upper incisor teeth, multiple mediastinal and hilar lymph nodes, bilateral lung metastases, and thrombi in the left inferior pulmonary veins extending into the atrium and left superior pulmonary veins with a bilateral moderate pleural effusion (Figure 3).

**Figure 4:**
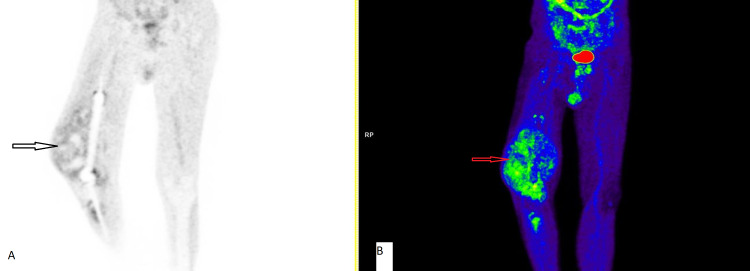
Positron emission tomography–computed tomography showing an orthopedic prosthesis in the knee region with a large lobulated fluorodeoxyglucose (FDG) avid lesion involving the right knee joint region with areas of necrosis and calcification. Seen with Maximum Intensity Projection (MIP) black & white image (A)  and colored image (B).

A large soft tissue lesion with areas of necrosis and calcification within the lesion was observed; it measured 14 × 12 × 17 cm in the right thigh and knee joint (Figure 4).

In view of the large swelling and severe pain, the patient was offered the option of palliative amputation of the right lower limb. However, this was refused by the patient, who was then lost to follow-up. The patient succumbed to metastatic chondrosarcoma and its complications in November 2020.

Written informed consent was provided by the next of kin to use the patient’s information for academic and publication purposes.

## Discussion

Chondrosarcoma is a malignancy of the cartilaginous tissue of the bone. It accounts for 15-20% of all primary bone tumors, with the median patient age at presentation being 30-50 years [11]. Chondrosarcomas are mainly classified as conventional chondrosarcomas of Grades 1-3. Ninety percent of all chondrosarcomas are conventional, and of these, 90% are Grades 1-2 tumors. A Grade 1 chondrosarcoma is called an atypical cartilaginous tumor. The remaining 10% of chondrosarcomas are classified as de-differentiated, mesenchymal, and clear cell variants [11]. Chondrosarcomas are also classified as primary or central (arising from the medullary cavity) and secondary (arising from the periosteum) chondrosarcomas. The most common site of origin is the pelvic bone followed by the ribs, femur, humerus, spine, scapula, and tibia. Sixty-five percent of all chondrosarcomas harbor *IDH1* or *IDH2* mutations, whereas *CDKN2A* and *COL2A1* alterations are also found in few cases [11]. Alterations in the retinoblastoma pathway have been detected in the majority of mesenchymal, de-differentiated, and clear cell-type chondrosarcomas. Extra-skeletal myxoid chondrosarcomas are classified as soft tissue sarcomas associated with typical t(9;22) (q22;q11-12) or t(9;17) (q22;q11) chromosomal translocations [11].

Symptoms include mild-to-moderate pain depending upon the anatomical location and size of the tumor. Wide excision of the tumor with the achievement of negative margins is the treatment of choice. Radiation therapy is indicated for patients with borderline resectable or unresectable tumors, for those who underwent incomplete resection, and for palliation of symptoms in those with advanced disease. Conventional chondrosarcoma is traditionally chemo-resistant with a minimal role of chemotherapy as adjuvant treatment or in those with metastases. Lungs are the most common site of metastasis. Other sites of metastasis include the liver, brain, kidneys, heart, and skin [1-4]. The findings of some small studies have indicated the role of oral drugs like pazopanib, dasatinib, and the IDH1 inhibitor ivosidenib [12-14]. De-differentiated and mesenchymal chondrosarcomas should be treated like osteosarcoma and Ewing’s sarcoma, respectively [15].

In patients presenting with oral cavity metastasis, the primary malignancies commonly involve the breasts, lung, thyroid, kidney, skin, and gastrointestinal tract. Lung and breast cancers are the most common primary malignancies in affected males and females, respectively [1-4].

Patients with oral mucosal metastases present a diagnostic dilemma. They usually present to dentists with oral lesions, the differential diagnoses of which may include pyogenic granulomas, peripheral giant cell granulomas, capillary hemangiomas, and fibromas that need to be ruled out.

The prognosis of such patients is poor with a reported two-to-nine-month survival duration. Our patient succumbed within two months of this rare presentation. There have been only six reported cases of chondrosarcoma with metastases to the oral cavity. Out of these, three had gingival metastases without bony involvement. Our patient constituted the fourth such case of gingival metastasis without bony involvement. 

The unique clinical features in this case include the presence of bulky lung metastasis with bilateral pleural effusion, gingival metastasis without bony involvement, and the short survival period after the patient presented with gingival metastasis. 

## Conclusions

Our experience with this patient shows the importance of detailed history-taking, thorough clinical examination, and considering metastasis as a differential diagnosis, even if there is no reported history of malignancy. Metastatic oral mucosal lesions do not show any pathognomonic signs on local examination, thus creating a diagnostic and therapeutic dilemma. These lesions may also be the first sign of an occult primary tumor, and maintaining an index of suspicion may prompt investigations that could help the clinician correctly diagnose and treat the underlying primary malignancy.
